# Combining alcohol interventions with tobacco addictions treatment in primary care—the COMBAT study: a pragmatic cluster randomized trial

**DOI:** 10.1186/s13012-017-0595-7

**Published:** 2017-05-18

**Authors:** Nadia Minian, Dolly Baliunas, Laurie Zawertailo, Aliya Noormohamed, Norman Giesbrecht, Christian S. Hendershot, Bernard Le Foll, Jürgen Rehm, Andriy Samokhvalov, Peter L. Selby

**Affiliations:** Centre for Addiction and Mental Health, 100 Stokes Street, Toronto, ON M6J1H4 Canada

**Keywords:** Alcohol, Tobacco, Cancer prevention, Health care practitioner, Primary care, Clinical decision support system

## Abstract

**Background:**

Tobacco and alcohol use present multiplicative risk for aerodigestive cancers. Reducing alcohol consumption improves smoking cessation outcomes and reduces cancer risk. Risky alcohol consumption and smoking are often treated separately despite concurrent treatment potentially leading to better outcomes for each. However, no rapidly scalable program exists for combined interventions in primary care clinics spread across wide geographic areas. This cluster randomized trial aims to report on the effects of a novel clinical decision support system (CDSS) on intervention rates by primary care practitioners addressing risky alcohol use in a smoking cessation program.

**Methods/design:**

We will be implementing a clinical decision support system (CDSS) in 221 primary care sites participating in the Smoking Treatment for Ontario Patients (STOP) program across Ontario, Canada. Sites will be blindly allocated to one of two clinical decision support systems guiding practitioners to provide a risky alcohol use intervention to smokers attempting to quit using nicotine replacement therapy (NRT). Risky alcohol use is defined as drinking above the Canadian Cancer Society’s low-risk drinking guidelines. Primary analysis will measure the proportion of risky drinkers offered an alcohol intervention in each CDSS arm at baseline. Patients will be contacted by phone or email to track smoking cessation and alcohol consumption rates at 6- and 12-month follow-up.

**Discussion:**

Upon completion of the trial, the effect of different clinical decision support systems on practitioner behaviour, and on client tobacco and alcohol use, will be discussed. If the CDSS successfully promotes SBIRT for risky alcohol use in a primary care setting and/or improves patient-level outcomes, including smoking cessation rates and alcohol use reduction, this tool can be used as a model for other web-based behaviour change interventions integrated into primary care practice.

**Trial registration:**

ClinicalTrials.gov NCT03108144

## Background

In the coming decades cancer is set to become a major cause of morbidity and mortality in every region of the world [1]. The evidence indicates that both tobacco use and alcohol consumption are independently major preventable risk factors [2]. Tobacco use increases the risk of developing at least 14 types of cancer and accounts for about 25–30% of all deaths from cancer, including 87% of deaths from lung cancer [2]. Alcohol consumption is causally related to cancers of the oral cavity, pharynx, larynx, oesophagus, liver, colon, rectum, and female breast [3]. Alcohol use accounts for about 3 and 10% of total cancers diagnosed in women and men, respectively, and of that, 40–98% occurs due to alcohol consumption greater than the recommended guidelines [4, 5]. Furthermore, the combined use of alcohol and tobacco confers a multiplicative, positively associated risk for aerodigestive and other related cancers [6–14]. For example, the combined effect of tobacco and alcohol has been shown to produce a population-attributable risk (PAR) of 85% for hypopharyngeal/laryngeal cancer, demonstrating that 85% of cases would be eliminated if concurrent alcohol consumption and smoking had never occurred [15]. Similarly, co-use of alcohol and tobacco explains a large proportion of oropharyngeal (PAR = 74%), esophageal (PAR = 67%), and oral cancers (PAR = 61%) [15].

There is substantial high-quality evidence that shows the efficacy of Screening, Brief Intervention, and Referral to Treatment (SBIRT) to reduce hazardous drinking [16–23]. However, few health care practitioners have implemented brief alcohol interventions into their daily routine despite the short time commitment (5–10 min) [24]. Furthermore, excess alcohol consumption and smoking are often treated separately; although, reducing or stopping drinking when quitting smoking and vice versa may lead to better outcomes [25–28]. With a lack of clear pathways, health care practitioners may be less likely to address alcohol consumption in smoking cessation treatments, thus presenting a missed opportunity for cancer prevention.

There is potential to address this gap through implementation of just-in-time clinical decision support systems (CDSSs). A CDSS is an interactive computer program designed to assist health care practitioners by automating simple decision-making processes enabling efficiency in the workplace to improve patient care [29–33].

This study aims to assess whether the addition of a web-based CDSS—designed to prompt practitioners in real time to conduct SBIRT with patients who are drinking above recommended guidelines—influences the proportion of practitioners delivering a brief intervention to their eligible patients quitting smoking.

In this paper, we describe the protocol for a cluster randomized trial named “Personalized patient alerts and care pathways to prompt prevention interventions for combined alcohol and tobacco users in primary care” or COMBAT. The COMBAT trial will be operationalized via the Smoking Treatment for Ontario Patients (STOP) program, an established smoking cessation program implemented at the primary care level in Ontario, Canada. The STOP program offers up to 26 weeks of smoking cessation treatment, consisting of nicotine replacement therapy and behavioural counseling, at no cost to the patient.

## Methods/design

### Participants

Primary health care clinics in Ontario, Canada, implementing an existing smoking cessation program—the STOP program—at the time of the study are eligible to participate. The vast majority of three types of primary health care clinics across Ontario implement the STOP program: 150 out of 184 family health teams (FHTs), 59 out of 67 community health centres (CHCs), and 17 out of 23 nurse practitioner-led clinics (NPLCs). Clinics enroll their patients into the STOP program using a centralized, online portal; the STOP portal is an online data management and collection tool used by all clinics to complete participant enrollment, record smoking status at each visit, monitor treatment, and receive real-time progress reports. This tool allows for processes to be streamlined and for health care practitioners to track their patients’ progress throughout the program. All those FHTs, CHCs and NPLCs operational in the STOP program as of March 14, 2016 (222 clinics), are eligible for randomization, except one known to use paper-based patient enrollment options exclusively (i.e. any clinic not using the online portal). In total, 221 clinics will be randomized into the COMBAT trial.

To build capacity of all practitioners implementing the STOP program, an interactive webinar will be presented on SBIRT implementation using CDSS, as web-based SBIRT training has shown to be effective for and favourable among primary care practitioners [34]. The webinar audience will include the STOP Community of Practice (*n* = 300) of implementers, physicians, and executive directors, who interact through bi-weekly teleconferences to communicate updates, clarify procedures, address barriers or gaps in program delivery, and share experiences with other practitioners.

### Trial design

The COMBAT study will be a cluster randomized controlled trial with clusters (i.e. primary care clinics in Ontario, Canada) randomized to one of two treatment arms. Health care practitioners working in clinics randomized to the intervention arm, group B, will receive computer alerts when a patient is identified as consuming alcohol above the recommended guidelines and will be guided to provide the patient with a brief intervention and an educational workbook. Practices randomized to the treatment-as-usual arm, group A, will have access to the same resources available to practitioners in group B but will not receive computer alerts.

The web-based CDSS will take advantage of a platform that overcomes the common barriers to system-wide implementation, such as expensive time-consuming programming changes and validation for each electronic medical record (EMR) system, and will have features that are associated with improvement, such as clinical workflow integration, decision support at time of care, and electronic and direct recommendations [35].

### Randomization

Clinics will be the unit of randomization. Two stratification factors will be defined: clinic type with three levels (FHT, CHC, and NPLC) and clinic size with two levels, resulting in six strata. Actual clinic size, measured as number of trial participants enrolled, is not directly observable a priori; therefore, expected clinic size will be estimated based on past STOP enrollment. Independently for each clinic type, the two levels of clinic size will be set such that expected total enrollment in the two levels will be balanced. Within each stratum, clinics will be randomly allocated in a 1:1 allocation ratio to control (group A) or intervention (group B). Treatment allocation (randomization) for each clinic will be determined for all operational clinics en masse. Any clinics that begin implementing the STOP program after the randomization cut-off date (April 11, 2016) will not be eligible for participation in the COMBAT trial. The random assignment of treatment to clinic will be computer generated using the ralloc command of statistical computer software Stata V.13 [36].

### Blinding

This pragmatic, cluster trial is designed to evaluate an intervention to change practitioners’ behaviour. Blinding of the clinic via its practitioners will therefore not be possible as the practitioner will be aware of the presence or absence of the portal prompting system.

Participating clinics will not be told of their treatment allocation until the trial begins. Investigators are blinded to the randomization arm. Data analysis will be blinded to treatment allocation.

### Patient screening

Participants in the COMBAT trial will be enrollees of the existing STOP smoking cessation program and will therefore be cigarette smokers wishing to quit, or those who have already begun their quit attempt.

Upon enrollment in the STOP program, participants will complete a baseline enrollment survey about their tobacco use and related measures. Alcohol consumption will be measured on the baseline survey using a seven-day timeline followback (TLFB) questionnaire [37] and the Alcohol Use Disorders Identification Test (AUDIT) [38]. Women consuming seven or more and men consuming fourteen or more alcoholic beverages (13.6 g per standard drink) in the past week—as measured by the TLFB—as well as any individual endorsing any episode of consuming five or more drinks on one occasion (AUDIT item 3) will be considered to be exceeding the Canadian Cancer Society (CCS)’s cancer prevention alcohol consumption guidelines [39]. In order for a participant to be eligible for the COMBAT trial, their baseline enrollment survey must be administered by a practitioner, in English, into the online portal in real time, so that the clinical interaction can be supported by the CDSS. Therefore, those patients who are administered the baseline survey on paper, or in French, cannot be included in the trial.

Patient consent for participation in the STOP smoking cessation program will be obtained at the time of enrollment. Individuals younger than 18 years old will enroll with the consent of their legal guardian if required to do so.

### Brief intervention and referral to treatment

In clinics assigned to group B (intervention), practitioners will receive an automated decision aid tool (CDSS) embedded in the STOP program’s online portal. The tool will provide decision support as part of the STOP program’s clinical workflow in three steps. In the first step, the tool will automatically score screening tool responses and identify to practitioners, in the form of a pop-up message, those patients who reported consuming alcohol in excess of CCS cancer prevention guidelines. In the second step, the tool will prompt practitioners to provide a brief alcohol reduction or abstinence intervention; the tool will provide guidance on how to conduct a brief intervention, or allow practitioners to conduct an intervention of their own, or to decline to conduct an intervention at that time. In the third step, practitioners will be asked to provide the patient with the appropriate educational, alcohol resource.

The trial will assess the effect of an online portal-based computer decision support system on health care practitioners’ provision of educational resources addressing alcohol consumption (i.e. “Referral to Treatment” portion of SBIRT) to patients who are exceeding cancer prevention alcohol drinking guidelines and engaging in an attempt to quit smoking. We designed two educational resources, one encouraging alcohol reduction, and one encouraging alcohol abstinence. Practitioners will be encouraged to deliver the alcohol reduction resource to patients exceeding CCS cancer prevention alcohol consumption guidelines. The alcohol reduction resource was adapted from four evidence-based resources including the National Institute on Alcohol Abuse and Alcoholism’s “Rethinking your Drinking” [40]; BC Partners for Mental Health and Addictions’ “Problem Substance Use Workbook” [41]; Capital Health Nova Scotia’s “My Choice: A Workbook For Making Changes” [42]; and College of Family Physicians of Canada and Canadian Centre on Substance Abuse’s “Drinking Smart: Your Health and Alcohol Consumption” [43]. In order to ensure the resources reflect the needs and interests of the population of interest, the adaptations were shaped by a community-informed engagement event with clients enrolled in the STOP program who reported drinking alcohol above the Canadian Cancer Society’s low-risk drinking guidelines.

However, participants who exceed cancer prevention alcohol consumption guidelines may be alcohol dependent, in which case, advice to abstain from alcohol consumption is warranted [44]. For participants scoring above a brief screen (AUDIT-C) cut-off (female >2 points; male >3 points), the online portal will launch a mandatory, full AUDIT (AUDIT-10) screen for alcohol dependence risk [38]. Practitioners will be trained to advise participants scoring above the AUDIT-10 cut-off (20 points) to abstain from alcohol consumption and to provide the alcohol abstinence resource including contact information for a provincial referral system (http://www.connexontario.ca/) for further treatment at local addiction programs.

In clinics assigned to group A (control), practitioners will administer the same alcohol screening tools and have access to the same educational resources as group B. However in order to identify patients who report consuming alcohol in excess of CCS cancer prevention guidelines, they will have to score patients’ screening tool responses (as they will not receive prompts from the portal) and decide which educational resource is the most appropriate for their patient.

**Fig. 1:**
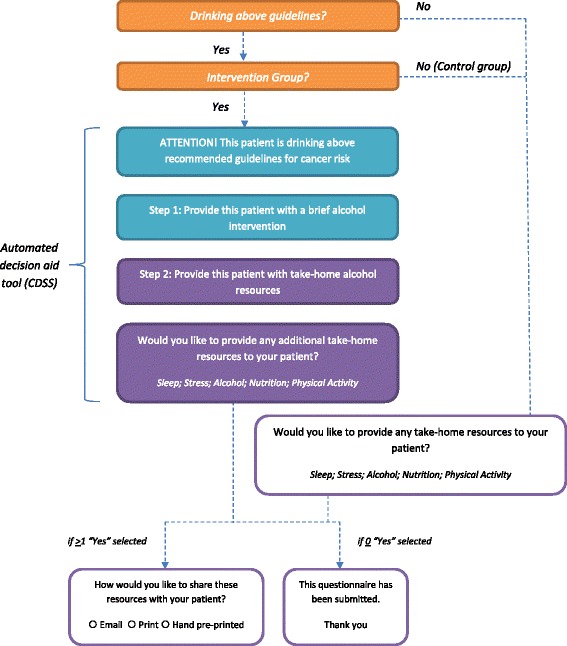
Study workflow diagram

Refer to Fig. 1 for a visual depiction of the study work flow.

All patients will be contacted to complete a six-month follow-up survey, where they will be asked about their current drinking and smoking behaviours.

### Outcomes

The primary outcome of the study, measured at the cluster level, will be the offer of an appropriate educational alcohol resource, reduction or abstention, to patients exceeding the cancer prevention alcohol consumption guidelines upon completion of STOP smoking cessation program enrollment. This dichotomous outcome is recorded by the STOP program’s online portal for each enrolling participant.

Given that patients can refuse practitioners’ offer of resources, the secondary outcome of the study, measured at the cluster level, will be the acceptance of the educational alcohol resource by patients exceeding the cancer prevention alcohol consumption guidelines. This dichotomous outcome is also recorded by the STOP program’s online portal for each enrolling participant.

The tertiary outcome, measured at the patient level, will be patient abstinence from smoking, and alcohol consumption within cancer prevention guidelines, at the 6-month follow-up survey as recorded in the STOP program’s online portal. Smoking abstinence will be defined by a negative response to the seven-day point prevalence question, “Have you had a cigarette, even a puff, in the last 7 days?” Alcohol consumption at follow-up will be measured using the same survey items as those used in the baseline enrollment survey, described previously. Alcohol consumption within CCS guidelines will be defined as the inverse of alcohol consumption in excess of CCS guidelines.

### Effect size

Based on measures of alcohol consumption used in the STOP Program prior to the start of the COMBAT trial, and accounting for intra-cluster correlation within each clinic as well as unequal enrollment by clinic, we assumed an enrollment of 23 participants per clinic over the 12-month course of this trial, in 189 clinics, for a total of 4347 subjects exceeding CCS guidelines, with enrollment size varying by a coefficient of 1.04. We set alpha = 0.05 and power = 80% and estimated an intra-cluster correlation coefficient of 0.04. Since there were no available estimates of the trial’s primary outcome measure prior to the initiation of the trial, the minimum detectable effect size was estimated for a range of values of *P*_1_, the proportion of control group enrollments in which practitioners delivered the brief intervention. The estimated detectable effect size ranged from a relative risk of 1.45 (if *P*_1_ = 0.1) to 1.04 if (*P*_1_ = 0.9).

An interim analysis in the form of a power calculation was conducted at the 8-month mark once estimates of *P*1 (defined above) were available. As described above, we used a method that accounts for intra-cluster correlation within each clinic as well as unequal enrollment by clinic, now incorporating the updated measures of alcohol consumption introduced with the start of the trial. The observed average cluster size was 12.71 and cluster sizes varied by a coefficient of 1.38. Setting alpha = 0.05, the analyst, unblinded to treatment allocation, obtained an estimate of *P*_1_ from the data collected to that date. To detect a relative risk of 1.20, the required sample size was estimated to be 6158. Therefore, the recruitment period will be extended until such a time as that sample size is achieved.

### Statistical methods and analysis

Descriptive statistics will compare cluster-level and patient-level baseline characteristics between the two treatment groups.

Results will be analyzed using a generalized estimation approach for fitting logistic regression using a population-averaged method with treatment arm and stratification variables (i.e. organization type and cluster size) regarded as fixed effects and clinic (i.e. cluster) as random effects in the model. To account for clustering, an exchangeable correlation matrix and robust standard errors will be specified [45]. The intra-cluster correlation coefficient will be estimated, and if it is found to be negative, an analysis not adjusting for clustering will be used.

The following baseline variables will be treated as potential confounders: gender; age; body mass index; identifying as First Nations, Inuit or Métis; household income; employment status; educational attainment; depression; AUDIT-C score; heaviness of smoking index; marijuana use; opioid use; treatment for mental health problem; drug treatment; and comorbid conditions. To identify the covariates to be included in the statistical models for the primary, secondary and tertiary analyses, we will fit separate models including each covariate one at a time. The final model will include those covariates such that their inclusion changes the estimated treatment effect by at least 10%. Decisions about the inclusion of covariates will not be based on statistical significance (i.e. *p* values).

As the primary and secondary outcomes will be captured by the online portal, complete case analyses will be used (i.e. imputation will not be used for these outcomes). The tertiary outcome will be available only for those patients who complete a 6-month follow-up survey; therefore, missing data is expected. A single imputation of best-case scenario (i.e. not smoking and drinking within guidelines) and a single imputation of worst-case scenario (i.e. smoking and drinking in excess of guidelines) will be conducted. If the best-case and worst-case scenarios imply different conclusions, a multiple imputation approach that accounts for the clustered structure of the data will be employed.

All analyses will follow an intention-to-treat approach in which clusters and participants are analyzed in the intervention arm to which they were originally assigned.

Qualitative interviews are taking place with health care practitioners to examine the facilitators and barriers of implementing SBIRT within their practice, as well as the diffusion of knowledge to others within their practice. We will analyze the interviews using a hybrid approach of inductive and deductive coding [46].

### Ethical approval and trial status

The study was reviewed by the Research Ethics Board at the Centre for Addiction and Mental Health (approval number: 035-2015). At the time of manuscript submission, the trial is ongoing with baseline data and primary and secondary outcome data collection in progress. The process evaluation data collection has not yet been undertaken.

### Retrospective trial registration

Initially, this study was believed not to qualify as a clinical trial as per the World Health Organization definition because the primary outcome measures health care practitioner behaviour change, rather than patient health outcomes. However, at the time of manuscript submission, study investigators learned it was appropriate to register any trial involving human participants as a clinical trial. Since this study’s tertiary outcome is to measure patient-level behaviour change, this study was retrospectively registered with ClinicalTrials.gov (NCT03108144). To eliminate any risk of reporting or publication bias, all study investigators have remained blinded to preliminary data analyses, which are conducted by an analyst external to the investigator team to monitor study progress.

## Discussion

This novel web-based platform for prevention interventions could shift the current practice of addressing alcohol and tobacco separately in the same patient to one where it is feasible for practitioners to provide a combined intervention for both behaviours using an evidence-informed computer-guided intervention. This program may also overcome the barrier of low implementation rates of brief interventions for alcohol use in primary care by automating best practices for cancer prevention.

By the end of this study, we will have intervened with 221 primary care clinics, which in turn will have the opportunity to intervene with approximately 4800 smokers who drink above CCS guidelines.

An important strength of this protocol is the use of a cluster randomized trial design to test a clinically important question for primary care sites in Ontario. In the field of implementation science, this study examines an innovative, web-based strategy to facilitate the uptake of evidence-based intervention practices in a primary care setting. The approach is pragmatic (as defined by PRECIS-2) [47], highly valuing external validity. Future studies will be able to use knowledge gained from this study to model computer-facilitated, just-in-time decision support strategies for other types of clinical encounters especially those requiring behaviour change such as nutrition and weight loss, or management of diabetes or hypertension.

The use of a web-based portal overcomes issues related to incompatibility across various electronic medical records in primary care settings. The permanent addition of an alcohol intervention to the STOP web-based portal would lead to a system-wide implementation of integrated alcohol and tobacco care pathways at relatively low cost. Therefore, scaling up is possible at low cost due to high adoption of the platform. In the field of health systems research, findings from this study can inform future planning and integration of other behavioural interventions—such as physical activity and mood management—into primary care settings. This program can also be scaled out beyond primary care to Addiction Agencies and Public Health Units.

Some potential limitations should be acknowledged. As noted earlier, health care practitioners will not be blinded to the treatment allocation. During this study, a health care practitioner might work in two different clinics, one assigned to group A (control) and one to group B (intervention). In this case, there is the possibility of contamination of knowledge; the health care practitioner might apply knowledge from the reminders received while working in a clinic assigned to group B while working in a clinic assigned to group A. This possible contamination could decrease the trial effect and lead to a more conservative effect estimate. However, we anticipate that this will be a rare occurrence. Another limitation is that we are unsure if we will be powered to detect any significant difference for our tertiary outcome before the study concludes. However, like most studies, our a priori sample size calculation was based on being powered to detect a significant difference in our primary outcome.

## Abbreviations

AUDIT: Alcohol Use Disorders Identification Test
CCS: Canadian Cancer Society
CDSS: Clinical decision support system
CHC: Community health centres
COMBAT: Personalized patient alerts and care pathways to prompt prevention interventions for combined alcohol and tobacco users in primary care
FHT: Family health team
NPLCs: Nurse practitioner-led clinics
SBIRT: Screening, brief intervention, and referral to treatment
STOP: Smoking Treatment for Ontario Patients
